# Piwil2 is reactivated by HPV oncoproteins and initiates cell reprogramming *via* epigenetic regulation during cervical cancer tumorigenesis

**DOI:** 10.18632/oncotarget.11810

**Published:** 2016-09-01

**Authors:** Dingqing Feng, Keqin Yan, Ying Zhou, Haiyan Liang, Jing Liang, Weidong Zhao, Zhongjun Dong, Bin Ling

**Affiliations:** ^1^ Department of Obstetrics and Gynecology, China-Japan Friendship Hospital, Beijing, China; ^2^ Department of Obstetrics and Gynecology, Anhui Provincial Hospital Affiliated to Anhui Medical University, Hefei, China; ^3^ Department of Gynecology and Oncology, Anhui Provincial Cancer Hospital, Hefei, China; ^4^ School of Medicine, Tsinghua University, Beijing, China

**Keywords:** Piwil2, cell reprogramming, tumor initiating cell, HPV oncoprotein, cervical cancer

## Abstract

The human papillomavirus (HPV) oncoproteins E6 and E7 are risk factors that are primarily responsible for the initiation and progression of cervical cancer, and they play a key role in immortalization and transformation by reprogramming differentiating host epithelial cells. It is unclear how cervical epithelial cells transform into tumor-initiating cells (TICs). Here, we observed that the germ stem cell protein Piwil2 is expressed in pre-cancerous and malignant lesions of the cervix and cervical cancer cell lines with the exception of the non-HPV-infected C33a cell line. Knockdown of *Piwil2* by shRNA led to a marked reduction in proliferation and colony formation, *in vivo* tumorigenicity, chemo-resistance, and the proportion of cancer stem-like cells. In contrast, *Piwil2* overexpression induced malignant transformation of HaCaT cells and the acquisition of tumor-initiating capabilities. Gene-set enrichment analysis revealed embryonic stem cell (ESC) identity, malignant biological behavior, and specifically, activation targets of the cell reprogramming factors *c-Myc*, *Klf4*, *Nanog*, *Oct4*, and *Sox2* in *Piwil2*-overexpressing HaCaT cells. We further confirmed that E6 and E7 reactivated *Piwil2* and that E6 and E7 overexpression resulted in a similar gene-set enrichment pattern as *Piwil2* overexpression in HaCaT cells. Moreover, *Piwil2* overexpression or E6 and E7 activation induced H3K9 acetylation but reduced H3K9 trimethylation, which contributed to the epigenetic reprogramming and ESC signature maintenance, as predicted previously. Our study demonstrates that *Piwil2*, reactivated by the HPV oncoproteins E6 and E7, plays an essential role in the transformation of cervical epithelial cells to TICs *via* epigenetics-based cell reprogramming.

## INTRODUCTION

Recently, the PIWI family of proteins has emerged as new players in the transcriptional and post-transcriptional regulation of gene expression [1-5]. The members of the PIWI subfamily are characterized by highly conserved PIWI and PAZ domains [3] and play crucial roles for gametogenesis, stem cell self-renewal, and epigenetic regulation in various organisms [6, 7]. Four members of the PIWI subfamily (Piwil1, 2, 3, and 4) have been identified in the human genome. Among them, Piwil2 is mainly expressed in testis or embryonic cells among normal tissues but is widely expressed in various types of tumors, including prostate, gastrointestinal, breast, cervical, ovarian and endometrial cancer in humans and in breast tumors, rhabdomyosarcoma and medulloblastoma in mice [8-15]. The underlying mechanism of Piwil2 in tumorigenesis may differ among various cancer cells, such as anti-apoptosis by activation of the Stat3/Bcl-XL pathway [9], suppressing P53 expression by enhancing Stat3 phosphorylation [16], promotion of proliferation *via* the Stat3/cyclin D1 pathway [9, 10], or increasing c-Myc expression by facilitating NME2 binding to the G4-motif [5]. In particular, Piwil2 is predominantly expressed in cancer stem cells (CSCs) and in precancerous stem cells (pCSCs) [10, 11, 17-19], indicating that it might play an important role in tumor initiation.

The formation of malignant tumors includes a lengthy, reversible pre-cancerous stage, which may naturally regress or progress. Piwil2 is ectopically activated in certain stages of pre-cancerous lesions of various organs [17, 20-22], suggesting that Piwil2 expression is an early event in the process of cell transformation caused by carcinogens or inflammatory cytokines. Cervical carcinoma develops from pre-neoplasia through a multistep process. High-risk human papillomavirus (HR-HPV) is the major cause of cervical cancer and its precursor stages of cervical intraepithelial neoplasia (CIN, graded 1-3 according to severity). CIN1 lesions are mild dysplasias that mainly spontaneously regress, whereas CIN2/3 lesions are severe dysplasias that are likely to progress if untreated. Previous studies from our group and others have demonstrated that Piwil2 is expressed in cervical CSCs from cervical cancer patients as well as in cervical cancer cell lines [11, 17, 18]. Piwil2 promotes proliferation and inhibits apoptosis in tumor cells [9, 15, 23]; however, the underlying mechanisms remain largely unclear.

In this work, we sought to expand knowledge of Piwil2 expression during cervical cancer tumorigenesis. Our study reveals that Piwil2 activates multiple germline factors, such as *c-Myc*, *Sox2*, *Nanog*, *Oct4*, and *Klf4*, through epigenetic programming and subsequently reprograms somatic cells into tumor-initiating cells (TICs), thus offering a potential therapeutic target for patients with cervical cancer and especially CIN lesions.

## RESULTS

### Piwil2 expression in cervical cancer and its precursor stages

**Figure 1:**
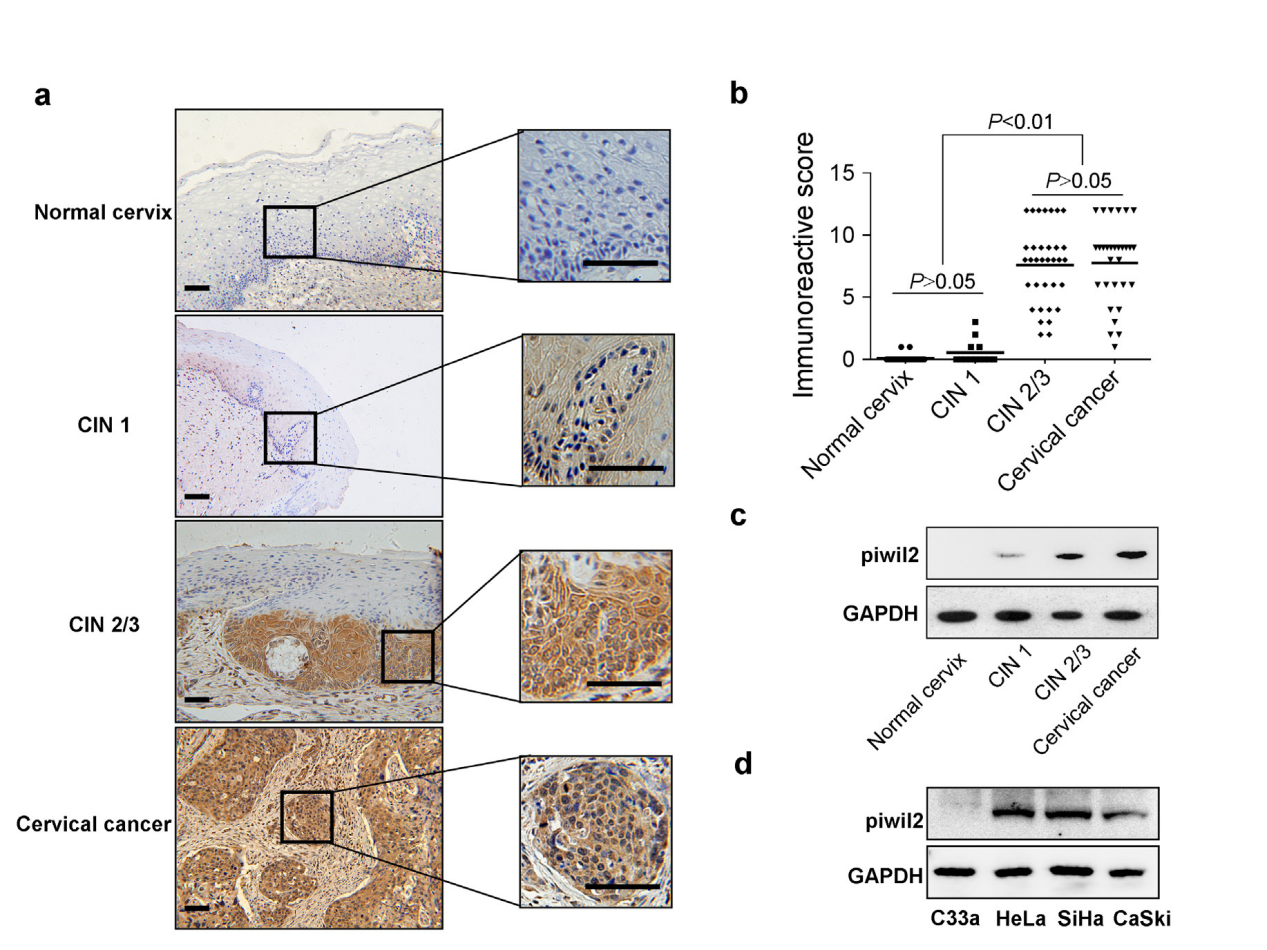
Characteristic Piwil2 expression in cervical cancer and its precursor stages **a.** Piwil2 expression detected by IHC in CIN lesion and cervical cancer tissue. Scale bar, 100 μm. **b.** Immunoreactive score of Piwil2 staining in CIN lesions and cervical cancer tissue. **c.** Western blot analysis of Piwil2 expression in CIN lesions and cervical cancer tissue. GAPDH expression is presented as a loading control. **d.** Western blot analysis of Piwil2 expression in cervical cancer cell lines.

Piwil2 is stably expressed in pCSCs and CSCs [17], which strongly suggests that Piwil2 is expressed in pre-cancerous lesions and malignant lesions. To explore the histological patterns of Piwil2 expression throughout cervical cancer development, we first measured Piwil2 expression by immunohistochemistry (IHC) analysis in formalin-fixed paraffin-embedded samples derived from cervical lesions (Figure 1a and 1b). No Piwil2 expression was observed in the basal layer of the normal cervix epithelium, whereas lesion cells in CIN2/3 and cervical cancer exhibited positive cytoplasmic and nuclear staining (Figure 1a). Only 4 of 13 CIN1 cases weakly expressed Piwil2 in the cytoplasm, and there was no significant difference in the immunoreactive score in the CIN1 tissues compared with the normal cervix tissues (Figure 1b). Western blot (WB) analysis also revealed a consistent characteristic Piwil2 IHC expression in specimens from patients with cervical lesions (Figure 1c). In HeLa, SiHa, and CaSki cervical cancer cell lines, which contain an integrated HR-HPV genome of type 18, 16, or both, respectively, Piwil2 expression was observed; however, in the C33a cell line, which is negative for HR-HPV DNA and RNA, no expression was detectable (Figure 1d). These results suggest that reactivation of Piwil2 in cervical cancer may be related to the integration of HR-HPV DNA into the host cell genome.

### Piwil2 inactivation confers tumor-suppressing effects

**Figure 2:**
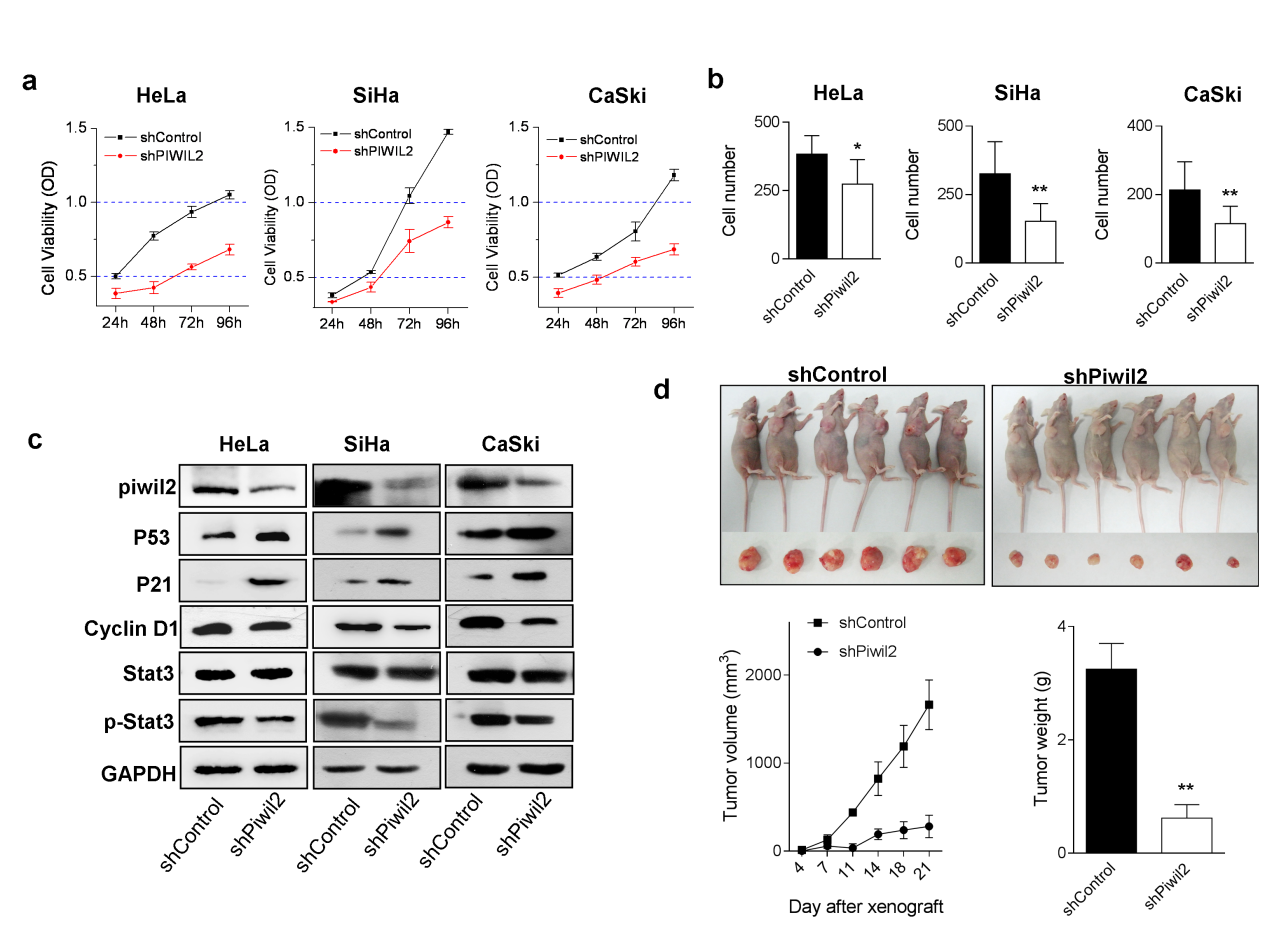
Piwil2 knockdown affects cervical cancer cell line proliferation, invasion, and *in vivo* tumorigenicity **a.** HeLa, SiHa, and CaSki cells were stably transfected with control shRNA or Piwil2 shRNA, and cell viability was measured daily. **b.** Numbers of invading cells in clones stably transfected with control shRNA and Piwil2 shRNA. **c.** Equal amounts of lysates from cancer cell lines stably transfected with control shRNA or Piwil2 shRNA were separated by SDS-PAGE, and proteins were analyzed by western blotting with specific antibodies against Piwil2 and molecules regulating cell proliferation. **d.** Tumor growth over time was measured after the subcutaneous injection of 5×10^6^ of SiHa cells stably transfected with shPiwil2 control shRNA and Piwil2 shRNA. Tumor volume was monitored by caliper measurements twice weekly, and tumor weight was measured after sacrifice at the end of the experiment. The data are presented as the mean±SD. *P < 0.05 and ** *P* < 0.01 by Student's *t*-test.

Because Piwil2 promotes proliferation and inhibits apoptosis in tumor cells [23], it is rational to exploit the tumor-suppressing effects of cervical cancer therapeutic approaches. Notably, when Piwil2 was knocked down, the proliferation and invasion of cervical cancer cell lines were significantly inhibited (Figure 2a and 2b). WB analyses confirmed that Piwil2 knockdown led to an up-regulation of P53 and P21 but a significantly decreased level of p-Stat3 and cyclin D1 (Figure 2c), which, to some extent, contributed to the cell proliferation inhibition. To further verify the *in vivo* antitumor effects by targeting Piwil2, SiHa cell lines stably transfected with shRNA were injected subcutaneously into the oxters of nude mice. Tumors were measured with calipers twice weekly, and the tumor volume was calculated as V = (length×width^2^)/2. After 3 weeks, the mean tumor volume for the shPiwil2 group was 280.98±127.69 mm^3^, whereas the tumor volume for the shControl group was 1662.53±280.98 mm^3^ (Figure 2d). Consistently with the tumor volume data, the mean tumor weights of the shPiwil2 and shControl groups were 3.25±0.45 g and 0.62±0.24 g, respectively (Figure 2d). Together, these results demonstrate that the knockdown of Piwil2 confers anti-tumor effects *in vitro* and *in vivo* in cervical cancer.

### Piwil2 induces somatic cell malignant transformation

**Figure 3:**
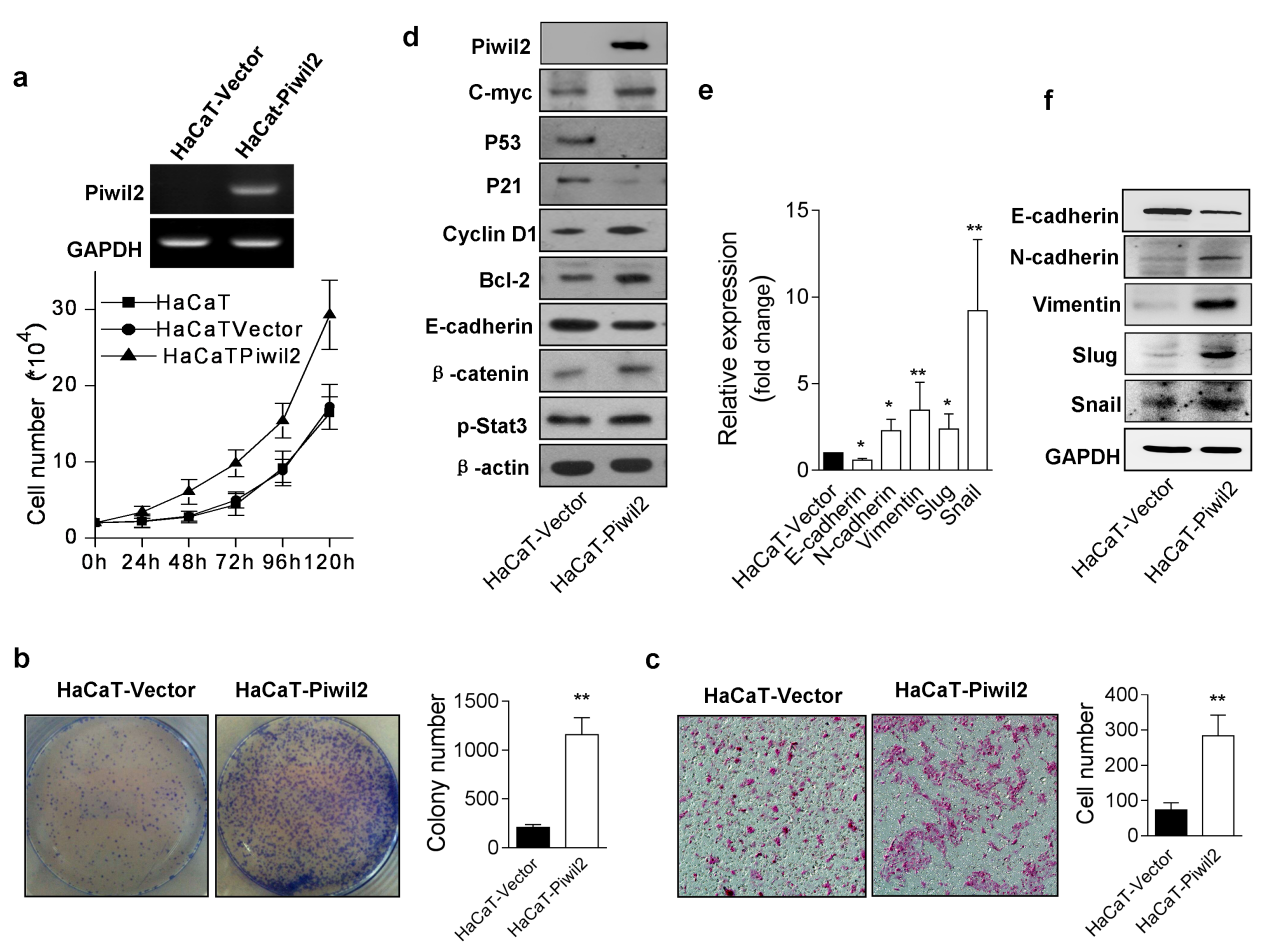
Piwil2 overexpression induces HaCaT cell malignant transformation **a.** HaCaT cells were transfected with lentivirus containing Piwil2 or lentiviral vector, and cell numbers were plotted daily. **b.**-**c.** Colony formation numbers and invading HaCaT cells infected with lentivirus containing Piwil2 or lentiviral vector. **d.** Proteins were analyzed by western blotting for c-Myc, E-cadherin, and molecules regulating cell proliferation and apoptosis. **e.**-**f.** The expression of EMT markers was determined by qRT-PCR and western blotting in HaCaT cells infected with lentivirus containing Piwil2 or lentiviral vector. The data are presented as the mean±SD. *P < 0.05 and ** *P* < 0.01 by Student's *t*-test.

Because Piwil2 is expressed in the pre-cancer stage of CIN2/3 and maintains the oncogenicity of cervical cancer cells, we sought to investigate whether Piwil2 plays an essential role in somatic cell malignant transformation. First, we detected the possible changes in cancer-associated characteristics caused by Piwil2. Expression lentivirus vectors were transfected into HaCaT cells, and stably transfected cells (HaCaT-Piwil2) were selected for experiments. Subsequently, cell proliferation, colony formation, and cell invasion were evaluated as previously described [24]. HaCaT cells overexpressing Piwil2 exhibited more rapid proliferation, greater colony formation, and enhanced invasive ability compared with the control HaCaT cells transfected with vector only (HaCaT-Vector) (Figure 3a, 3b, and 3c). To determine the potential underlying molecular mechanism, WB analysis revealed that c-Myc, p-Stat3, Cyclin D1, and Bcl-2 were upregulated, whereas the expression of P53 and P21 were significantly reduced (Figure 3d). Notably, the expression of the cell adhesion and epithelial marker, E-cadherin, decreased, whereas the expression level of β-catenin was enhanced (Figure 3d). The epithelial-mesenchymal transition (EMT) is an essential step in cell migration and invasiveness. After transfection with Piwil2, HaCaT cell lines exhibited phenotypic changes consistent with EMT, including E-cadherin downregulation and the acquisition of several mesenchymal markers (such as N-cadherin, Vimentin, Slug, and Snail) at both the mRNA and protein levels (Figure 3e and 3f).

**Figure 4:**
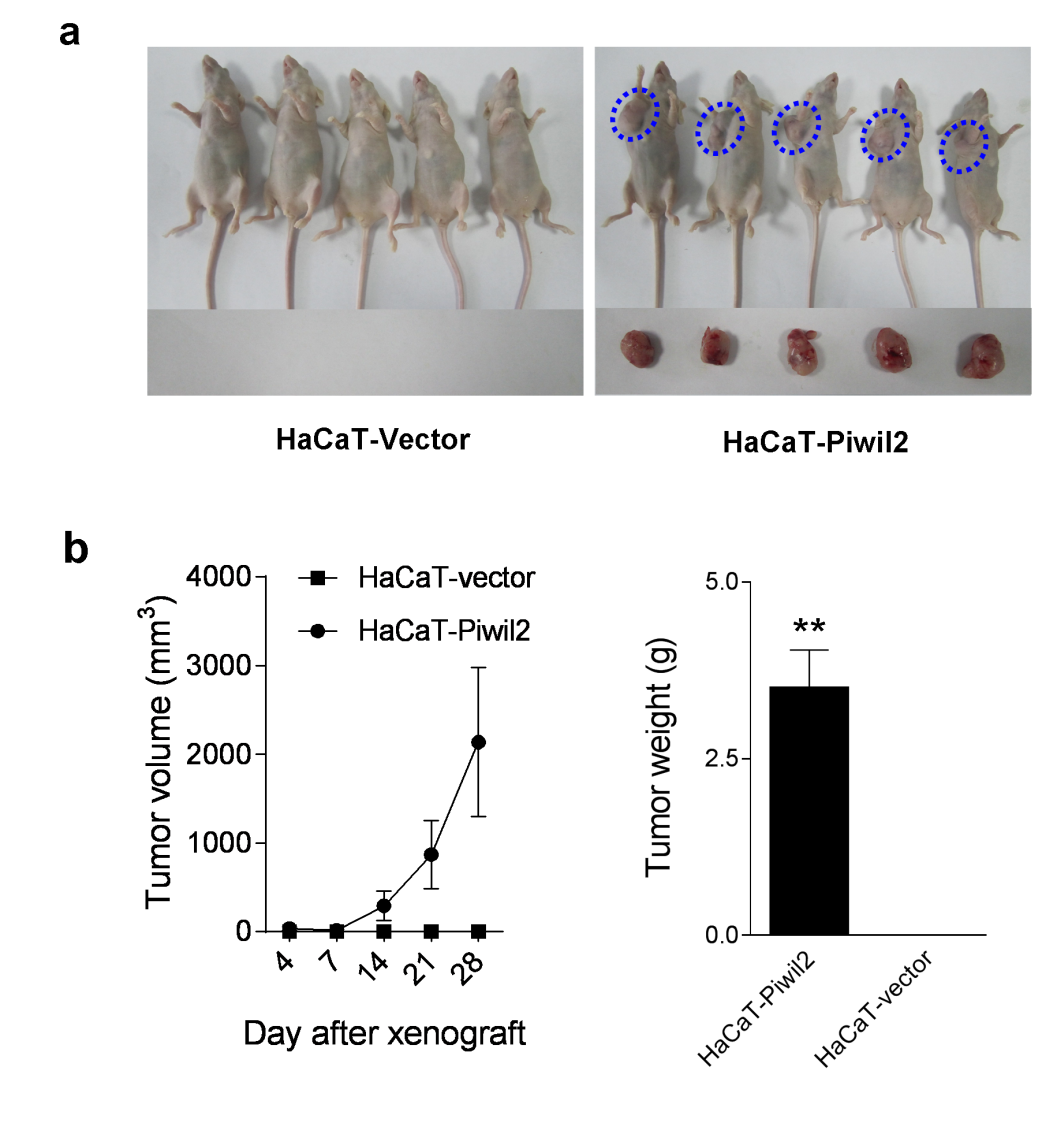
Piwil2 initiates tumorigenicity of HaCaT cells **a.** Approximately 5×10^6^ HaCaT cells transfected with Piwil2 or Vector were injected subcutaneously into the oxters of nude mice. Four weeks after injection, tumorigenesis in nude mice was observed. **b.** Tumor volume was monitored by caliper measurements twice a week, and tumor weight was measured after sacrifice at the end of the experiment. The data are presented as the mean±SD. ** *P* < 0.01 by Student's *t*-test.

Furthermore, the *in vivo* experiment demonstrated that Piwil2 promotes the tumorigenicity of HaCaT cells. HaCaT-Piwil2 cell lines formed tumors with a mean volume of 2137.63±838.90 mm^3^ 28 days after subcutaneous transplantation into the oxters of nude mice, whereas no tumor formation was observed when control HaCaT-Vector cells were used (Figure 4a and 4b).

Collectively, these findings suggest that Piwil2 expression promoted EMT and the acquisition of malignant biological behavior in HaCaT cells. We observed that forced Piwil2 expression induced, to some extent, the reprogramming of a small proportion of HaCaT cells to TICs.

### Piwil2 prompts the establishment of a cancer stem-like cell gene-set enrichment pattern

Cancer stem-like cells, which have been considered to be TICs, exhibit the ability to initiate and propagate tumors and exhibit gene expression signatures characteristic of embryonic stem (ES) cells [25]. Here, we analyzed the enrichment patterns of gene sets associated with cancer stem-like cell identity in the expression profiles of HaCaT-Piwil2 cells. We extracted some of these sets directly from published studies with a small change to their contents (Table S1), and some were extracted from the Molecular Signature Database (MSigDb) v5.0 of Gene Set Enrichment Analysis (GSEA) (http://software.broadinstitute.org/gsea/msigdb/index.jsp). We compiled 15 partially overlapping gene sets, which fell into three groups: (1) somatic cell reprogramming factors targets: Nanog, Oct4, Sox2 (NOS), c-Myc, and Klf4 bound and activated/co-activated the promoters of these gene sets. NOS TFs, a subset of NOS-activation targets, encoded transcription regulators [26]; (2) stem cell-expressed genes: three sets of genes overexpressed in stem cells according to some reports; and (3) malignancy-related pathways: five sets of genes involved in tumor cell proliferation, invasion, and metastasis.

**Figure 5:**
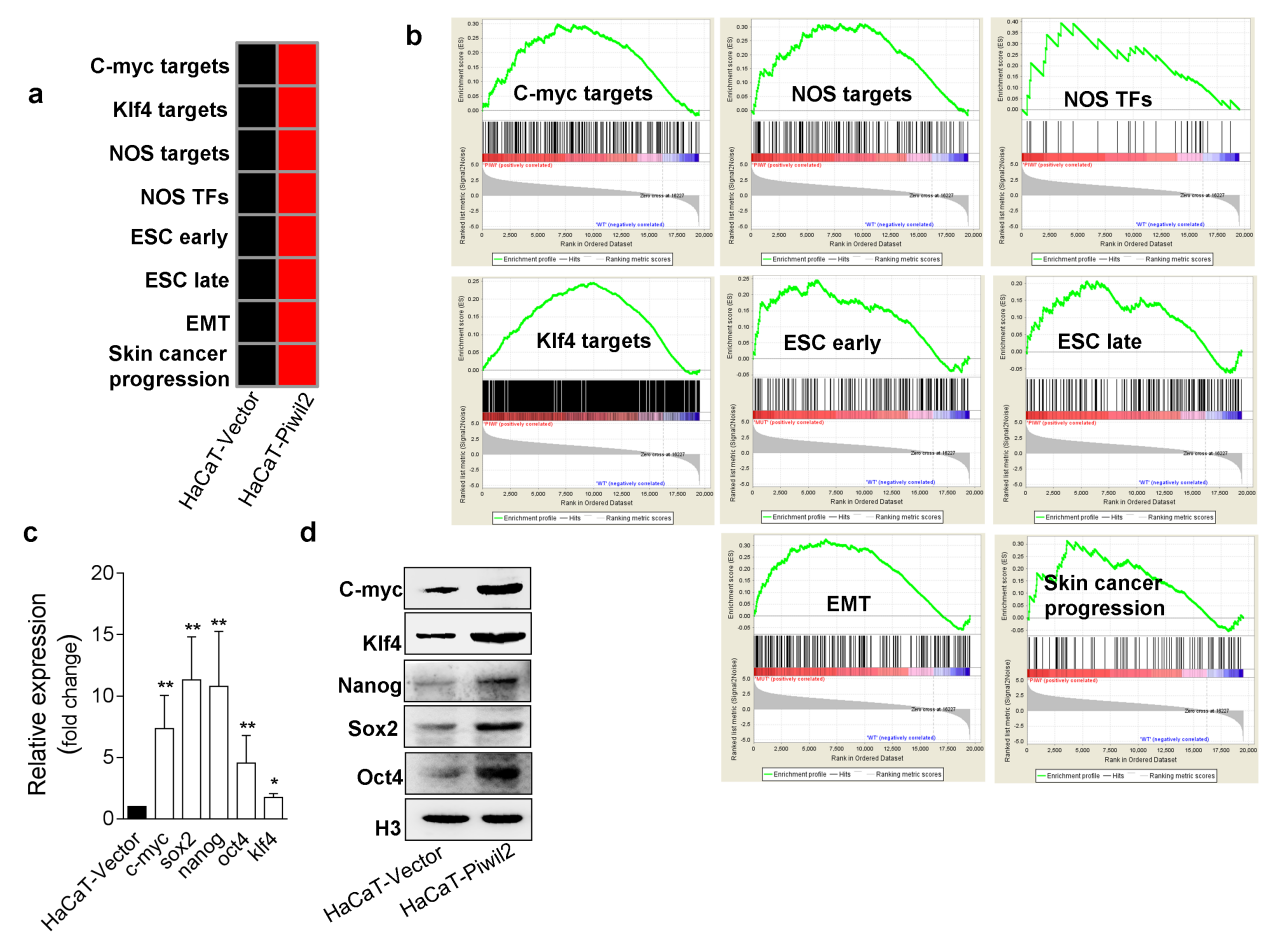
Piwil2 facilitates the establishment of a cancer stem-like cell gene-set enrichment pattern **a.** Gene-set enrichment in HaCaT cells transfected with lentivirus containing Piwil2 compared with cells transfected with lentiviral vector. Red, gene-set enrichment; black, no significant enrichment. **b.** GSEA analyses demonstrating the gene set expression and the relationship between target gene expression and factor occupancy. **c.**-**d.** The expression of 5 transcription factors associated with cell reprogramming was determined by qRT-PCR and western blotting in HaCaT cells transfected with lentivirus containing Piwil2 or lentiviral vector. The data are presented as the mean±SD. *P < 0.05 and ** *P* < 0.01 by Student's *t*-test.

We used GSEA to interpret the expression patterns of the 15 gene sets in microarray expression data. Fourteen gene sets were overexpressed in the HaCaT cells stably transfected with Piwil2 relative to the cells transfected only with vector (Figure 5a, 5b, and S1). The highest levels of gene-set enrichment were observed for the NOS targets and NOS TFs (Figure 5b). The upregulation of gene sets ESC and BOQUEST Stem cell exhibited a stem-like cell enrichment pattern in HaCaT-Piwil2 cells (Figure 5b and S1). Meanwhile, the levels of the gene sets for epithelial-mesenchymal transition (EMT), Jak-Stat3, Cyclin D1, and Skin cancer progression were also elevated in HaCaT-Piwil2 cells, indicating neoplasia transformation (Figure 5b and S1). We further confirmed that Piwil2 overexpression induced a significant upregulation of 5 “core” somatic cell reprogramming factors, *Nanog*, *Oct4*, *Sox2*, *c-Myc*, and *Klf4*, by quantitative RT-PCR (Figure 5c) and WB (Figure 5d). Overall, our findings indicate that Piwil2 may act as a key initial factor in the formation of cancer stem-like cells, which suggests that the cancer stem-like cell signature is reactivated to some extent during the course of tumor progression.

### The combination of E6 and E7 reactivate Piwil2 expression

**Figure 6:**
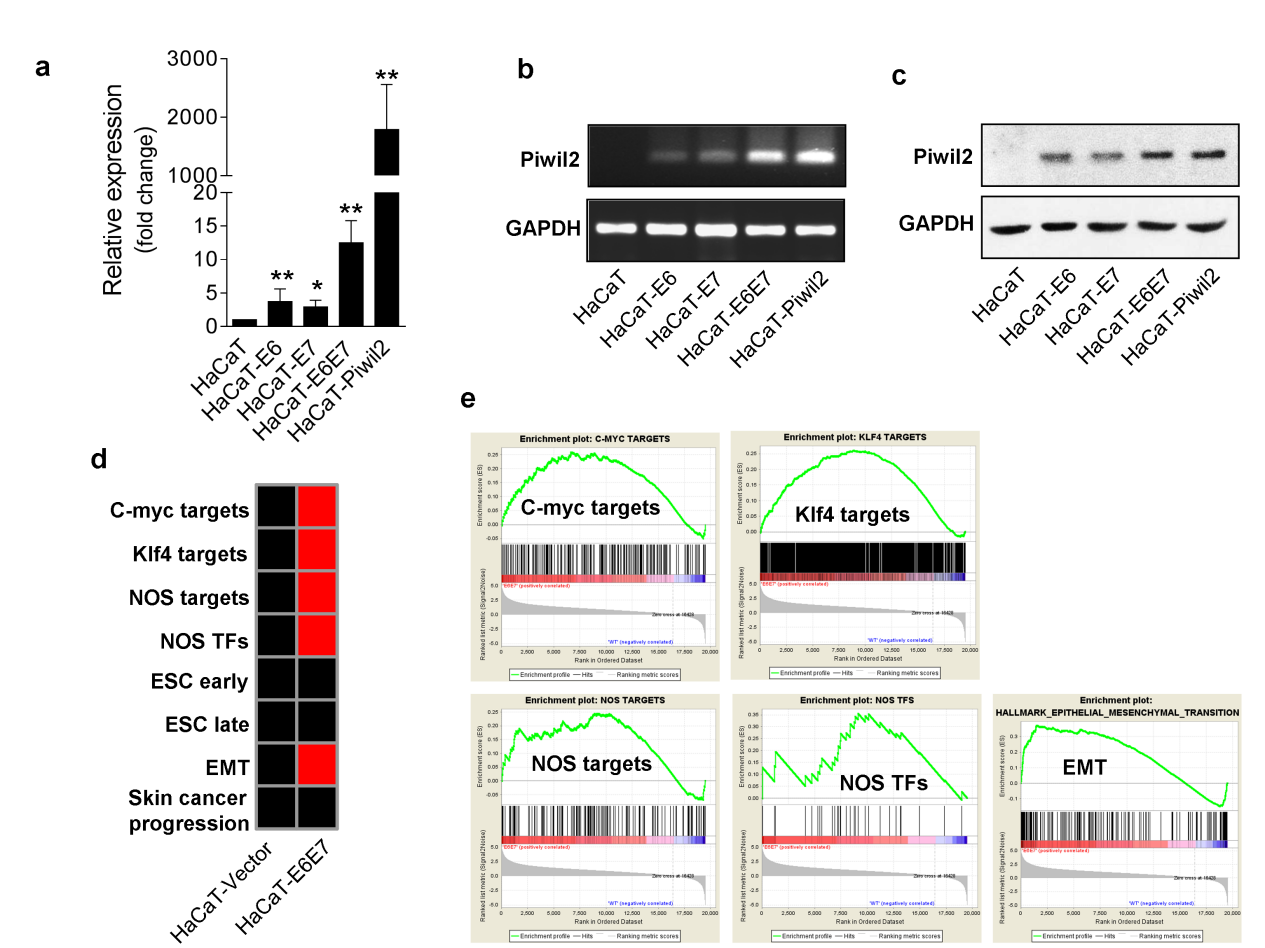
The HR-HPV oncoproteins E6 and E7 reactivate Piwil2 expression in HaCaT cells **a.**-**c.** qRT-PCR, RT-PCR and western blotting reveal upregulated expression of Piwil2 in HaCaT cells transfected with lentivirus containing complete HPV16 E6 and E7 sequences compared with those transfected with lentiviral vector. **d.** Gene-set enrichment pattern of HaCaT cells transfected with lentivirus containing complete HPV16 E6 and E7 sequences. Red, gene-set enrichment; black, no significant enrichment. **e.** GSEA analyses demonstrating the gene set expression and the relationship between target gene expression and factor occupancy. The data are presented as the mean±SD. *P < 0.05 and ** *P* < 0.01 by Student's *t*-test.

The HR-HPV oncoproteins E6 and E7 are the primary viral factors responsible for the initiation and progression of cervical cancer, and they act largely by inducing immortalization and transformation by reprogramming differentiating host epithelial cells to support viral DNA replication and cell division [27]. Our results also demonstrate that Piwil2 is expressed in HeLa, SiHa, and CaSki cells, which are positive for the integrated HR-HPV genome, whereas Piwil2 is undetectable in HPV-negative C33a cells (Figure 1d). Therefore, we investigated whether E6 and E7 can reactivate Piwil2 expression. First, HaCaT cells were transfected with the lentiviral vectors pLenti-HPV16E6-3×Flag-neo and pLenti-HPV16E7-3×Flag-neo individually or in combination. The stably transfected cells were selected with G418 and used for the detection of Piwil2 expression. WB and quantitative RT-PCR assays revealed that the combination of E6 and E7 exhibited a synergic effect in restoring Piwil2 expression, while either E6 or E7 led to slightly upregulated Piwil2 expression (Figure 6a, 6b, and 6c). Notably, GSEA of HaCaT-E6E7, compared with HaCaT-Piwil2, revealed a similar enrichment pattern of the gene sets of 5 “core” somatic cell reprogramming factors (*Nanog*, *Oct4*, *Sox2*, *c-Myc*, and *Klf4*) as well as EMT, Jak-Stat3, and Cyclin D1 (Figure 6d, 6e and S2), which contribute to the immortalization and transformation of cells overexpressing E6 and E7 [27, 28]. However, the ESC and BOQUEST Stem cell gene sets were not significantly upregulated relative to HaCaT cells transfected with vector (Figure 6d and S2).

### Piwil2 initiates cell reprogramming through epigenetic programming

**Figure 7:**
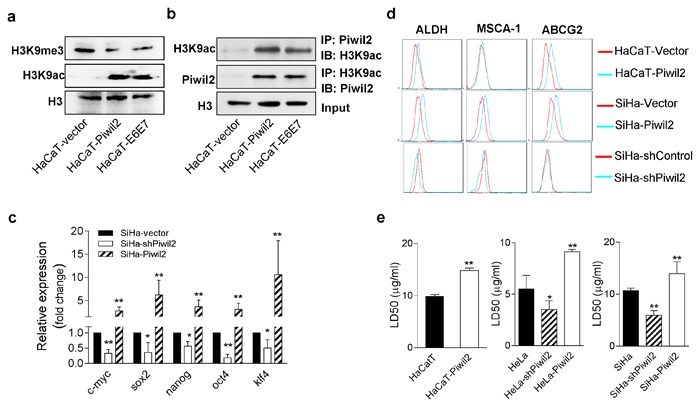
Piwil2 initiates cell reprogramming by regulating the balance between the acetylation and trimethylation of H3K9 **a.** Western blot showing significantly induced H3K9 acetylation but reduced H3K9 trimethylation in HaCaT cells transfected with Piwil2 or E6 and E7 compared with those only transfected with vector. **b.** Co-immunoprecipitation showing that Piwil2, either overexpressed or induced by E6 and E7, interacted with acetylated H3K9. **c.** EMT markers upregulated in SiHa cells exhibiting Piwil2 overexpression but downregulated in those cells in which Piwil2 was knocked down, as verified by qRT-PCR. **d.** The proportion of ALDH-, MSCA-1-, and ABCG2-positive cells, determined by FACS in cells with Piwil2 overexpression or knockdown. **e.** The LD50 dose of cisplatin, detected by CCK8 assay in cells with Piwil2 overexpression or knockdown. The data are presented as the mean±SD. *P < 0.05 and ** *P* < 0.01 by Student's *t*-test.

Previous studies have demonstrated that histone modification signatures, especially acetylation and trimethylation of histone 3 lysine 9 (H3K9), are important in controlling gene regulation in ESCs and TICs, in a process called epigenetic programming [20]. H3K9 acetylation (H3K9ac), an epigenetic mark associated with euchromatin, plays an important role in gene activation, thus maintaining “stemness”, by binding to the promoter or transcription start sites (TSS) of *NOS*, *c-Myc*, and *Klf4* [29]. Conversely, H3K9 trimethylation (H3K9me3), a mark of constitutive heterochromatin, acts as a silencer of transcriptional gene expression, and preventing methylation of H3K9 facilitates the production of induced pluripotent stem cells (iPS) [30]. In this study, we observed that Piwil2 overexpression in HaCaT cells significantly induced H3K9 acetylation but reduced H3K9 trimethylation relative to HaCaT cells transfected only with vector (Figure 7a). Furthermore, the same epigenetic modifications were also observed in HaCaT cells after E6 and E7 stable transfection (Figure 7a). Additionally, the results of co-immunoprecipitation (Co-IP) assays revealed that Piwil2, overexpressed in HaCaT-Piwil2 cells or induced in HaCaT-E6E7 cells, interacted with H3K9ac (Figure 7b). Most importantly, the forced expression of Piwil2 led to a clear upregulation of ALDH, MSCA-1, and ABCG2, three common markers of cancer stem-like cells [16], as detected by FACS (Figure 7d), and a significant LD50 increase of cisplatin, as determined by a CCK8 assay (Figure 7e). Moreover, in cervical cancer cells, Piwil2 overexpression also induced a significant upregulation of *c-Myc*, *Nanog*, *Oct4*, *Sox2*, and *Klf4* (Figure 7c), increased the proportion of cells that were ALDH, MSCA-1, and ABCG2 positive (Figure 7d) and subsequently increased cisplatin resistance (Figure 7e). In contrast, Piwil2 knockdown led to the significant downregulation of *c-Myc*, *Nanog*, *Oct4*, *Sox2*, and *Klf4* (Figure 7c) and slightly decreased the percentage of ALDH-, MSCA-1-, and ABCG2-positive cells, thus rendering cells more sensitive to cisplatin, as compared with cervical cancer cells transfected with the control vectors (Figure 7c, 7d, and 7e).

**Figure 8:**
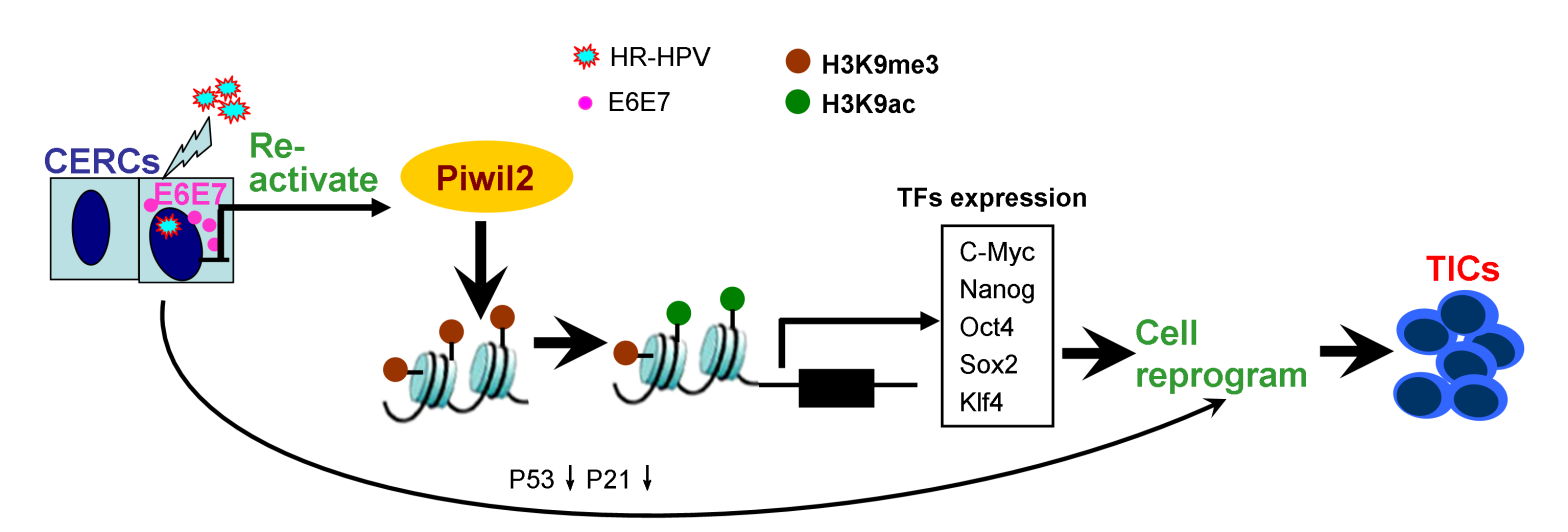
Model of cell reprogramming and tumor-initiating cell formation secondary to Piwil2 induction

Together, these findings reveal that Piwil2, whose expression is restored by the combination of E6 and E7 in cervical cancer, may reactivate the “core” somatic cell reprogramming factors *c-Myc*, *Nanog*, *Oct4*, *Sox2*, and *Klf4* by regulating the shift of acetylation and trimethylation of H3K9, thus initiating cell reprogramming and contributing to TIC formation (Figure 8).

## DISCUSSION

Cervical carcinoma progresses from CIN to invasive cancer through a multistep process. CIN1 is a histological diagnosis associated with continued viral replication and virus shedding and is not considered precancerous; in most cases, CIN1 spontaneously regresses. In contrast, CIN2/3, in which viral DNA has integrated into the host cell DNA, is substantially more likely to progress to invasive cancer and is currently regarded as the standard threshold for the treatment of precancerous lesions [31, 32]. In parallel the dynamic histopathological process, in this study, the IHC results revealed that the cytoplasm and nuclei in CIN2/3 and cervical cancer specimens showed positive staining for Piwil2, whereas only 4 out 13 CIN1 cases weakly expressed Piwil2 in the cytoplasm but not in the nuclei, and no Piwil2 was expressed in normal cervical samples. In conjunction with reports that Piwil2 is specifically expressed in pCSC and CSC [17], these data suggest that Piwil2 is a potential diagnostic and prognostic marker for cervical lesions as well as a key molecule initiating tumorigenesis. However, little is known regarding how normal cells are reprogrammed into TICs.

The demonstration that adult fibroblasts can be reprogrammed into pluripotent ES-like cells upon the forced expression of three or four (or more) factors, including *Klf4*, *Oct4*, *Sox2*, *Nanog*, and c*-Myc* [33, 34], raises the possibility that the combined “atavic expression” of these pluripotency factors and specific oncogenes may also induce TIC transformation from somatic cells under the cellular stress of specific carcinogens. In this study, our results revealed that forced expression of Piwil2, a stem-cell protein, in HaCaT cells induced the significant upregulation of *c-Myc*, *Klf4*, *Nanog*, *Oct4*, and *Sox2*. We further analyzed the enrichment pattern of 15 gene sets in the HaCaT cells stably transfected with Piwil2. Notably, HaCaT-Piwil2 cells not only exhibited an ES-like enrichment pattern but also significantly upregulated proliferation-associated gene sets and malignancy-related pathway gene sets. In parallel with these signatures, HaCaT-Piwil2 cells, which had acquired tumor-initiating capability, expressed the TIC markers ALDH, MSCA-1, and ABCG2 and generated highly aggressive tumors when injected into mouse oxters. Our data demonstrate that Piwil2 is sufficient to induce TIC formation through reactivating transcription factors *c-Myc*, *Klf4*, *Nanog*, *Oct4*, and *Sox2* as well as their target gene sets, thus inducing somatic cell reprogramming.

The HR-HPV oncoproteins E6 and E7 are the primary viral factors responsible for the initiation and progression of cervical cancer [27, 35]. E6 proteins degrade the tumor suppressor P53 and activate the transcription of telomerase reverse transcriptase, which in conjunction with RB inactivation by E7, are essential for malignant transformation [27, 35]. Importantly, the P53/P21 pathway has been shown to act as a critical barrier to the cell reprogramming process, and suppression of P53 and its downstream target P21 significantly increases the reprogramming efficiency [36-38]. Our data indicate that overexpression of Piwil2 also significantly suppressed the expression of P53 and P21 in HaCaT cells; in contrast, Piwil2 knockdown led to an upregulation of P53 and P21 in cervical cancer cell lines. Furthermore, in this study, we demonstrated that E6 and E7 restored Piwil2 expression, sequentially reactivated gene sets associated with cancer stem-like cell identity, and induced TIC formation. However, HPV-immortalized cells are not tumorigenic in nude mice [39] and require extensive passaging in tissue culture or the coexpression of additional oncogenes, such as v-ras or v-fos, to acquire the ability to form tumors [27, 40, 41]. These data indicate that the quantity and intracellular location of Piwil2 expression is the key determinant for the progression of TICs to cancer cells, which parallels the histological progression of CIN lesions to malignant lesions.

In addition to the five somatic cell reprogramming factors and their targets noted above, we also observed two epigenetic modifications, H3K9ac and H3K9me3, by WB and Co-IP assays. In both mouse and human ESCs, H3K9ac is enriched at gene promoters and is highly correlated with gene expression and with various genomic features, including different active histone marks and pluripotency-related transcription factors [42-44]. In contrast, increased H3K9 methylation mediates global chromatin silencing and ESC differentiation, and demethylation of H3K9me3 increases the transcriptional expression of the pluripotency genes *Nanog*, *Oct4*, and *Sox2* [29, 30]. Consistently with these data, our results demonstrated that Piwil2 overexpression or induction by E6 and E7 significantly increased the level of H3K9ac and reduced H3K9me3, which led to the upregulation of the cancer stem-like cell gene-sets. Furthermore, the co-IP results revealed an interaction between Piwil2 and H3K9ac, suggesting a possible positive feedback loop in this epigenetic programming.

In summary, the current study demonstrates that Piwil2 reactivates multiple germline factors, including *c-Myc*, *Klf4*, *Sox2*, *Nanog*, and *Oct4*, through epigenetic programming and subsequently reprograms somatic cells into TICs. Notably, this process may be initiated by oncoproteins E6 and E7 in high-risk HPV, thereby contributing to cervical carcinogenesis. Therefore, targeting Piwil2 may be an effective therapeutic option for patients with cervical cancer and CIN lesions in particular.

## MATERIALS AND METHODS

### Ethics statement

The investigation was conducted in accordance with the ethical standards of Declaration of Helsinki and has been approved by the Ethics Committee of Anhui Medical University. Written informed consent was obtained from each patient. Approval for experiments involving animals was granted by the Committee on the Use and Care of Animals of Anhui Medical University.

### Patients and samples

Formalin-fixed paraffin-embedded cervical tissues were selected from the archives of the Department of Pathology of Anhui Provincial Hospital affiliated with Anhui Medical University. The clinical stages were determined by two certified gynecologists according to the modified International Federation of Gynecology Obstetrics (FIGO) system for cervical cancer published in 2000. The pathologic diagnosis of these specimens was evaluated by at least two pathologists according to the 2003 revised criteria proposed by the World Health Organization. The specimens included 23 cases of normal cervical tissues, 13 cases of CIN1, 34 cases of CIN2/3, and 30 cases of cervical cancer. The normal cervical tissues were obtained from patients who underwent a hysterectomy for reasons other than neoplasia of the cervix and had no history of CIN. The cervical neoplasm specimens were obtained from patients who underwent an operation or cervical biopsy. None of the patients had received any tumor-specific therapy before surgical excision.

### Immunohistochemistry staining and evaluation of immunoreactivity

Immunohistochemistry staining and evaluation of immunoreactivity were performed as described previously [45]. Briefly, paraffin blocks of the specimens were cut into 5-μm slices and then processed using standard deparaffinization and rehydration techniques. The slices were stained with an anti-Piwil2 antibody (ab85084, Abcam, UK) and sequentially visualized using the ChemMate Detection Kit (SV0002, Boster, China). Immunoreactivity was semiquantitatively evaluated on the basis of staining intensity and distribution using an immunoreactivity score as follows: intensity score×proportion score. The intensity score was defined as follows: 0, negative; 1, weak; 2, moderate; or 3, strong. The proportion score was defined as follows; 0, negative; 1, < 10%; 2, 11-50%; 3, 51-80%; or 4, > 80% positive cells. The total score ranged from 0 to 12. The stained cervical tissues were scored by two researchers who were blinded to the clinical data.

### Cell culture and transfection

Human cervical cancer cell lines HeLa, SiHa, CaSki, C33a, and HaCaT were purchased from the American Type Culture Collection (ATCC) and cultured in DMEM (41965062, Gibco, USA) with 10% fetal bovine serum (16000044, Gibco).

To generate cell line overexpression Piwil2, HeLa, SiHa, CaSki, C33a, and HaCaT cells were transfected with lentiviral pLenti-CMV-Piwil2-SV40-EGFP or pLenti-EGFP as the control. To generate the stably transfected cell line HaCaT-E6E7, HaCaT cells were first infected overnight in 6-well plates with lentiviral pLenti-HPV16E6-3×Flag-neo and then infected with pLenti-HPV16E7-3×Flag-neo overnight after selection with 400 μg/mL G418 (A1720, Sigma-Aldrich, USA) for 8 days. Viral transfections were performed in the presence of 4 mg/mL polybrene (P4020, Sigma-Aldrich). To knock down Piwil2 expression, the human cervical cancer cell lines HeLa, SiHa, CaSki, and C33a were transfected in 6-well plates with 1 μg Piwil2 shRNA expression plasmids (TG302470, Origene, Beijing), which contained four unique 29-mer shRNA constructs, respectively (1. ATGAGGTTCGGCATGTTGAAGGACCATCA; 2. CCAAGATGGTGGTGTTTGTAGTTCAGAAG; 3. CATCAGGAGATTGTGGACAGCCTGAAGCT; and 4. TGGTATCAGCAGAGAAGTGGACAAGCCTC), or 1 μg scrambled sequence shRNA plasmid (TR30013, Origene) using Lipofectamine 3000 (L3000015, Invitrogen, USA). Cells stably expressing the shRNA were selected from transfected cultures with puromycin (9620, Sigma-Aldrich) for 2 weeks. The optimal concentration of puromycin for each cell line was determined by pre-experimental results.

### Cell proliferation assay

Cell proliferation assays using the WST-8 Cell Counting kit (CCK-8, Dojindo, Japan) per the manufacturer's instructions were performed in 96-well format in duplicate, and the assays repeated three times. The LD50 values were calculated by nonlinear regression analysis using GraphPad Prism software (San Diego, USA).

### Colony formation and cell invasion

The cells were seeded into 6-well plate at a density of 2000 cells per well after transfection. The medium was changed every three days. Approximately 2 weeks later, most of the cell clones contained more than 50 cells. The clones were washed with 1× PBS and stained with 0.05% crystal violet for approximately 10 min. Subsequently, the clones were imaged and quantified with Image-Pro Plus 6.0 (IPP6.0, Media Cybernetics, Inc.). Cell invasion was assessed using 24-well transwell plates (Corning, USA). Cells in the upper compartment of the chamber were suspended in DMEM with 1% fetal bovine serum, and the lower chamber contained DMEM with 20% fetal bovine serum. After a 24-h incubation, cells that passed through the Matrigel matrix were fixed and stained with haematoxylin and eosin and counted in 5 random microscopic fields.

### RT-PCR and qRT-PCR

Total RNA was extracted using TRIzol (15596018, Invitrogen) and converted to cDNA using Superscript III reverse transcriptase (2680, TaKaRa, Japan). The cDNA was amplified by PCR using the *Premix Ex Taq*™ (RR901, TaKaRa). Quantitative real-time PCR (qRT-PCR) was performed using the iQ™ SYBR^®^ Green Supermix Kit (170-8880, Bio-Rad, USA) on a CFX96 Touch™ real-time PCR instrument (Bio-Rad). The primer sets used in the RT-PCR and qRT-PCR are presented in Table S2.

### Antibodies and western blots

Antibodies specific to Stat3 (#9132, 1:1000), p-Stat3 (#9145, 1:2000), P21 (#2947, 1:1000), β-actin (#3700, 1:1000), N-cadherin (#13116, 1:1000), E-cadherin (#3195, 1:500), and GAPDH (#2118, 1:2000) were obtained from Cell Signaling Technology. Antibodies specific to Piwil2 (ab85084, 1:1000), histone H3 (ab10799, 1:1000), H3K9ac (ab10812, 1:1000), H3K9me3 (ab71604, 1:1000), c-Myc (ab32072, 1:500), Klf4 (ab72543, 1:1000), Nanog (ab80892, 1:500), Oct4 (ab181557, 1:1000), Sox2 (ab137385, 1:500), Vimentin (ab8978, 1:500), Slug (ab27568, 1:500), Snail (ab180714, 1:500), and β-catenin (ab32572, 1:5000) were purchased from Abcam. Antibodies specific to P53 (BM0102, 1:500), Cyclin D1 (BA0770, 1:1000), and Bcl-2 (BA0412, 1:200) were obtained from Boster Technology. Protein was prepared from the cells and tissues according to the kit manual (89900, Thermo, USA) except for the addition of an acid extraction step for histones [45]. After electrophoresis, the proteins were transferred to polyvinylidene fluoride (PVDF, IPFL00010, Merck Millipore, Germany) membranes and probed with the indicated primary antibodies. Incubation with species-specific secondary antibodies (Cell Signaling) was performed at room temperature for 1 hour. The blots were developed with chemiluminescent substrate (34080, Thermo), and autoradiography was performed with X-OMAT film (Kodak, Rochester, NY, USA).

### Immunoprecipitation (IP)

Cells were lysed with RIPA lysis buffer supplemented with protease inhibitor cocktail (04693124001, Roche, Germany). For Piwil2-IP, cell lysates were incubated with 2 μg of Piwil2-specific antibodies (ab36764, Abcam) or normal rabbit immunoglobulin G (IgG) as a negative control. For H3K9ac-IP, cell lysates were incubated with 2 μg of H3K9ac-specific antibodies (ab10812, Abcam) or normal rabbit IgG as negative control. After an overnight incubation at 4 °C, Protein G Agarose Beads were added (193258, Abcam) and continually incubated for 2 h. Immunoprecipitated proteins were then subjected to SDS-PAGE and western blot analysis.

### Microarray analysis of gene expression

Genome-wide mRNA microarray analysis was performed as described previously [24]. Gene expression data were analyzed with the Gene Set Enrichment Analysis (GSEA) software (http://software.broadinstitute.org/gsea/index.jsp) [46].

### Flow cytometry analysis

The cells were harvested and suspended with RPMI1640 containing 2% fetal bovine serum and stained with biotin-conjugated anti-human ABCG2 (13-8888, eBioscience, USA) for 30 min. After being washed twice in 1× PBS, the cells were stained with the following cocktail of markers: anti-human MSCA-1-APC, anti-human ALDH, and Streptavidin eFluor^®^450 (17-9669, 41-9595, 48-4317, eBioscience). Cells were then washed, resuspended in RPMI1640 with 2% fetal bovine serum, and analyzed by flow cytometry (FACSCalibur, BD). At least 10^6^ events were acquired and analyzed using FlowJo software.

### Establishment of tumor xenografts

*In vivo* experiments were performed in accordance with the institutional guidelines for the use of laboratory animals. Four-week-old female nude mice (CD1 *nu/nu* mice) were obtained from the Laboratory Animal Center of the Chinese Academy of Science (Shanghai, China) and maintained in a pathogen-free animal facility for at least 1 week before use. Approximately 5×10^6^ cells were injected subcutaneously into the oxters of nude mice. Tumors were measured with calipers twice weekly, and the volume was calculated as V = (length×width^2^)/2. At the end of the experiment, the mice were sacrificed, and the tumors were collected and weighed.

### Statistical analyses

The data are presented as the mean±SD. To assess the statistical significance of differences, *t* tests (SPSS software, version 13.0; SPSS Inc., USA) was performed. A *P* value < 0.05 was considered significant.

## SUPPLEMENTARY MATERIALS FIGURES AND TABLES






