# Supplementary material for: Piwil2 is reactivated by HPV oncoproteins and initiates cell reprogramming via epigenetic regulation during cervical cancer tumorigenesis
Source: Oncotarget. 2016 Sep 1;7(40):64575–88. doi: 10.18632/oncotarget.11810 (PMC5323100; doi:10.18632/oncotarget.11810)
Supplement: Supplementary file 1 [file oncotarget-07-64575-s001.pdf]

Piwil2 is reactivated by HPV oncoproteins and initiates cell reprogramming *via* epigenetic regulation during cervical cancer tumorigenesis

Supplementary Information

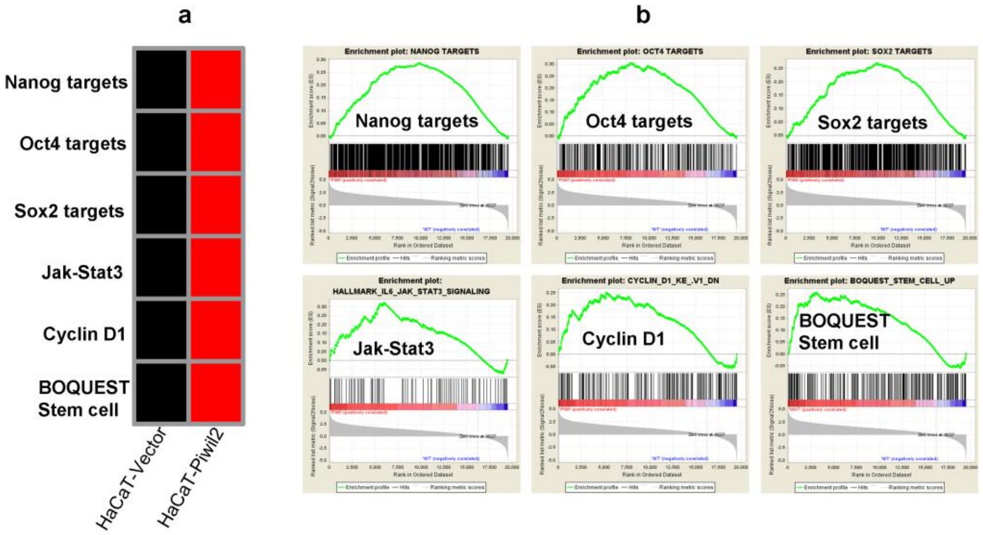

Figure S1: Gene-set enrichment pattern in HaCaT cells with Piwil2 overexpression

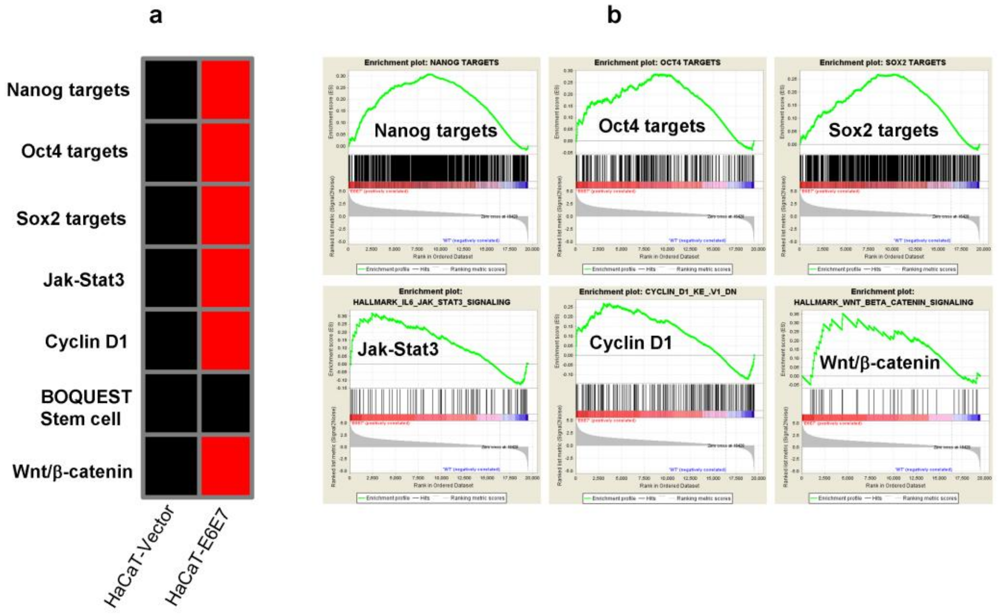

Figure S2: Gene-set enrichment pattern in HaCaT cells with E6 and E7 overexpression
