# Supplementary material for: Piwil2 is reactivated by HPV oncoproteins and initiates cell reprogramming via epigenetic regulation during cervical cancer tumorigenesis
Source: Oncotarget. 2016 Sep 1;7(40):64575–88. doi: 10.18632/oncotarget.11810 (PMC5323100; doi:10.18632/oncotarget.11810)
Supplement: Supplementary file 3 [file oncotarget-07-64575-s003.doc]

**Table S2. Specific primer sequences for RT-PCR and qRT-PCR**

| **Gene name** | **Forward primer sequences (5’-3’)** | **Reverse primer sequences (5’-3’)** |
| --- | --- | --- |
| Piwil2^†^ | GAGAATTCCTATGGATCCTTTCCGACCA | ACGTCGACTCACAGGAAGAACAGGTTCT |
| HPV16E6-3×Flag^†^ | ATGCACCAAAAGAGAACTGCAATG | TTACTTGTCATCGTCATCCTTGTAG |
| HPV16E7-3×Flag^†^ | ATGCATGGAGATACACCTACATTG | TTACTTGTCATCGTCATCCTTGTAG |
| β-actin^†^ | GTGGGGCGCCCCAGGCACCA | CTCCTTAATGTCACGCACGATTT |
| Piwil2 | TGCAGGCAGAGGCCATGTA | AGGCCTCGGAACATGGAGAC |
| E-cadherin | ACAATGCCGCCATCGCTTAC | AACTCTCTCGGTCCAGCCCA |
| N-cadherin | TGCCAGTGTGACTCCAACGG | GCCTGGCGTTCTTTATCCCG |
| Vimentin | AAGCAGGAGTCCACTGAGTA | GCTTCAACGGCAAAGTTCTC |
| Snail | TTCTCCTCTACTTCAGTCTCTTCC | GAGGTATTCCTTGTTGCAGTATTT |
| Slug | GCCCCATTAGTGATGAAGAGGAAA | AGCCCAGAAAAAGTTGAATAGGTC |
| c-Myc | GCAGCTGCTTAGACGCTGGA | CGCAGTAGAAATACGGCTGCAC |
| Klf4 | AAGAGTTCCCATCTCAAGGCACA | GGGCGAATTTCCATCCACAG |
| Nanog | CCTGTGATTTGTGGGCCTGA | CTCTGCAGAAGTGGGTTGTTTG |
| Oct4 | GTGCCGTGAAGCTGGAGAA | TGGTCGTTTGGCTGAATACCTT |
| Sox2 | GTGAGCGCCCTGCAGTACAA | GCGAGTAGGACATGCTGTAGGTG |
| GAPDH | CTTAGCACCCCTGGCCAAG | GATGTTCTGGAGAGCCCCG |

†, Primer sequences used for RT-PCR to amplify whole sequences of Piwil2, HPV16E6-3×Flag and HPV16E7-3×Flag.
